# Spinal Cord Injury and Pressure Ulcer Prevention: Using Functional Activity in Pressure Relief

**DOI:** 10.1155/2013/860396

**Published:** 2013-04-09

**Authors:** May Stinson, Rachel Schofield, Cathy Gillan, Julie Morton, Evie Gardner, Stephen Sprigle, Alison Porter-Armstrong

**Affiliations:** ^1^School of Health Sciences, Centre for Health and Rehabilitation Technologies, University of Ulster, Jordanstown BT37 0QB, Ireland; ^2^Health and Rehabilitation Sciences Research Centre, University of Ulster, Jordanstown BT37 0QB, Ireland; ^3^Belfast Health & Social Care Trust, Musgrave Park Hospital, Belfast BT9 7JB, Ireland; ^4^Centre for Assistive Technology and Environmental Access, Georgia Institute of Technology, Atlanta, GA, USA

## Abstract

*Background*. People with spinal cord injury (SCI) are at increased risk of pressure ulcers due to prolonged periods of sitting. Concordance with pressure relieving movements is poor amongst this population, and one potential alternative to improve this would be to integrate pressure relieving movements into everyday functional activities. *Objectives.* To investigate both the current pressure relieving behaviours of SCI individuals during computer use and the application of an ergonomically adapted computer-based activity to reduce interface pressure. *Design.* Observational and repeated measures design. *Setting.* Regional Spinal Cord Injury Unit. *Participants.* Fourteen subjects diagnosed with SCI (12 male, 2 female). *Intervention.*Comparing normal sitting to seated movements and induced forward reaching positions. *Main Outcome Measures.* Interface pressure measurements: dispersion index (DI), peak pressure index (PPI), and total contact area (CA). The angle of trunk tilt was also measured. *Results.* The majority of movements yielded less than 25% reduction in interface pressure compared to normal sitting. Reaching forward by 150% of arm length during an adapted computer activity significantly reduced DI (*P* < 0.05), angle of trunk tilt (p<0.05), and PPI for both ischial tuberosity regions (*P* < 0.001) compared to normal sitting. *Conclusion.* Reaching forward significantly redistributed pressure at the seating interface, as evidenced by the change in interface pressures compared to upright sitting.

## 1. Introduction

Pressure ulcers are one of the most common secondary complications of spinal cord injury (SCI) [1]. Increasing prevalence rates amongst individuals with SCI are attributed to repeatedly spending prolonged periods of time in the seated position coupled with limited mobility and sensation [2]. When sitting approximately 50% of body weight is concentrated over just 8% of body surface area, causing high interface pressure [3], consequently ischial tuberosity and sacral regions tend to suffer pressure ulceration the most [4, 5].

These wounds disrupt life, often causing episodes of both physical and financial disability [6, 7]. Pressure ulcer care has a significant impact on health care expenses, costing an annual *£*1.4–2.1 billion to the National Health Service [8]. Prevention on the other hand has been reported to cost approximately one-tenth of these treatment costs [9].

One of the most effective preventative methods in terms of cost and pressure relief is regular repositioning [10]. Within rehabilitation, individuals with SCI are taught and encouraged to perform regular repositioning movements in order to redistribute the build-up of pressure around the ischial tuberosity and sacral regions. These repositioning movements include vertical push-ups, lateral and forward leans. Although recommended as often as every 15–30 minutes [11], research has shown that concordance with regular repositioning is poor [12, 13].

Wheelchair users are reported to spend as much as 18 hours per day in their wheelchair, with many (54.7%) repositioning less often than once an hour [12, 13]. Therefore, strategies to encourage repositioning movements amongst SCI individuals warrant further exploration. One potential alternative to improving concordance with these traditional repositioning methods would be to integrate such movements within everyday functional activities to make them a more natural part of everyday life. 

A popular activity enjoyed by millions of people everyday is computer use. The Annual Communications Market Report [14] found that in the UK 8 out of 10 homes have internet access via a desktop or laptop computer. In 2011, internet users were reported to spend an average of 23.5 hours per month online. Furthermore, research has indicated that common occupations obtained by individuals with SCI include office, finance, clerical, administrative, technical, and professional jobs [15–17], all of which require some form of computer use. 

Physically, the demands of computer use are rated as one of the lowest amongst office tasks, requiring little body movement and exertion [18]. Thus it could be said that computer use, requiring a fairly static sitting posture, is a predominantly sedentary activity that consequently may increase the risk of pressure damage. 

Therefore, as computer use is a popular activity amongst individuals with SCI, used for both leisure and employment [19], and as computer use is thought to habitually restrict seated movement, the aims of this clinically based cohort study were twofold. The purpose of Strand A was to investigate the current pressure relieving behaviours of SCI individuals during everyday computer use, while Strand B aimed to investigate the application of an ergonomically adapted computer-based activity to reduce interface pressure. 

## 2. Methods

Recruitment and data collection took place in the Regional Spinal Cord Injury Unit, Musgrave Park Hospital, Belfast Health and Social Care Trust. Ethical approval was obtained from the Office of Research Ethics Committees, Northern Ireland, and research governance was provided by the Research and Development Office of the Belfast Health and Social Care Trust.

### 2.1. Sample

A convenience sample of 14 subjects diagnosed with spinal cord injury (12 male, 2 female; age range 23–62 years) was recruited to participate in this study (Table 1). Inclusion criteria required participants to be between 18 and 65 years of age, have a diagnosis of paraplegia or tetraplegia, and be current inpatients or outpatients of the Regional Spinal Cord Injury Unit, Musgrave Park Hospital, Belfast. Participants had to be able to safely perform a forward lean and be computer literate to a basic level to be included. Informed written consent was gained by the researcher on commencement of the study, and participants were informed that they could withdraw from the study at any time.

### 2.2. Instrumentation

The Xsensor X3 pressure mapping system, PX100:36.36.02 (Xsensor Technology Corporation, Calgary), consists of a thin, flexible pressure sensing mat and a hand held computer which graphically displays the distribution of interface pressure. The Xsensor pressure mapping mat was set at a recording resolution of 1 Hz and calibrated up to a maximum of 200 mmHg prior to each assessment.

The Activpal 3 accelerometer (PALTechnologies, Glasgow), a triaxial activity monitor which uses piezoelectric accelerometers to measure movement in three perpendicular axes, was used to measure direction of movement. This device was chosen because of its compact size (5 × 3.5 × 0.7 cms) and ability to respond accurately to gravitational and segmental movement acceleration [20] with a range of ±2 g and a sensitivity of 5 Hz when used as an inclinometer [21].

Participants used either a standard desktop computer or an iPad, depending on preference and fine motor skills. 

### 2.3. Experimental Protocol

Throughout both Strands A and B of the study, participants sat in their own wheelchair on their prescribed pressure relieving cushion and wore loose fitting clothing, for example, tracksuit bottoms. The Xsensor pressure sensing mat was placed between the participant and the wheelchair cushion surface. An Activpal3 accelerometer was attached to the participant's sternum [22] using the hydrogel PalStickies [21]. Participants positioned their wheelchair at the computer desk. The keyboard of the desktop computer was positioned with the home row (row beginning A, S, D, F) 19 cms from the front edge of the desk [23] in order to standardise the position for each participant. Similarly, the iPad was also positioned 19 cm from the front edge of the desk.

*Strand A.* Participants performed a self-selected computer-based activity for a one hour period. The frequency and type of repositioning movements performed throughout the one hour sitting period were determined by two methods: (1) through interface pressure mapping using the Xsensor pressure mapping system and by (2) the researchers' observational analysis of the type, timing, and frequency of participants' movements. Movements were categorised as either forward lean; left lean; right lean; push-up; other/task related movement such as typing or reading. Each movement was timed from the second posture change took place until a different posture was adopted. Interface pressure measurements for each movement were averaged and compared to periods of “normal upright sitting.” 

**Figure 1:**
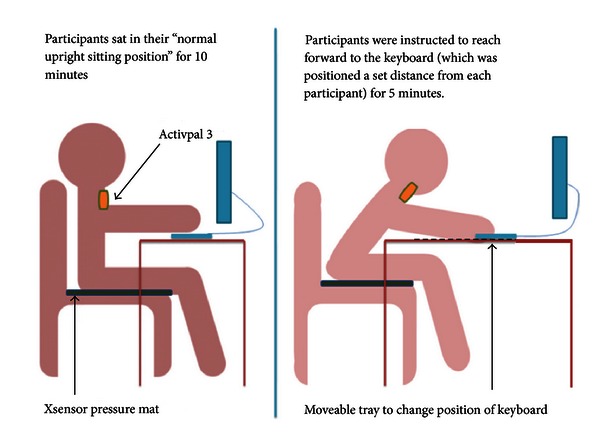
The positioning of equipment in Strand B.

*Strand B.* Participants positioned their wheelchairs at the desk as in Strand A; however, this time participants performed a computer-based activity of their choice for a 30 minute period, and the computer keyboard/Ipad was positioned on a moveable tray which alternated between two positions: (1) their normal upright sitting position and (2) a forward leaning configuration normalised to each participants arm length (150% × arm length measured from acromion process to 2nd metacarpophalangeal joint). Participants were instructed to sit in their “normal sitting position” and perform a computer activity of their own choice for a 10-minute period. The keyboard or iPad was then moved forward to the forward lean position. The participant was then instructed to reach to and hold this 150% position, whilst continuing their computer activity for a further 5-minute period. The computer keyboard/iPad was then moved back to the original position, and both the 10-minute “normal sitting” and the 5-minute forward lean trials were repeated. Measurement of interface pressure and trunk angle took place throughout this 30-minute period (Figure 1).

### 2.4. Outcome Measures

The primary outcomes of interest in this study were interface pressure and trunk angle. During both Strand A and Strand B, interface pressure measurements were investigated using the parameters of dispersion index, peak pressure index, and total contact area, which are defined as follows.

- *Dispersion Index* is the sum of the pressure distributed over the IT regions divided by the sum of pressure readings over the entire mat, expressed as a percentage.
- *Peak Pressure Index* is the highest pressure within a 9-10 cm^2^ area in the ischial region.
- *Total Contact Area* is the area of the sensors with pressures reading 10 mmHg or above.

The change in position of the participants' trunk during each movement was measured using an Activpal accelerometer-based sensor during both strands of the study.

Participant views of performing repositioning movements; current computer usage; and ease of performing the adapted activity were obtained using a short questionnaire at the end of Strand B.

### 2.5. Data Analysis

All data were analysed using SPSS software Version 17. Data recorded by the Activpal 3 accelerometer was converted into degree and direction of postural movement using an “Activpal2Posture” algorithm. (Activpal2posture configuration equation. Activpal3 reports ±2 g accelerations in units between 1–256. Orienting the Activpal using the human figure on the unit, the *x*-axis points downward, *y*-axis points left, and the *z*-axis projects forward out of the body plane. Using a custom script written in MATLAB, values from all three axes were converted into g units, and pitch angle was calculated using the equation: $\text{pitchangle}=\arctan(-z_{g},\sqrt{x_{g}^{2}+y_{g}^{2}})*180/\pi$, where *x*_*g*_, *y*_*g*_, and *z*_*g*_ represent accelerations in the respective axes.) 

*Strand A.* Pressure distribution patterns were visually examined to determine the frequency and type of movements. Observational analysis confirmed the type and duration of movements categorised as follows: forward leans; left lean; right lean; push-up; other or task related movements such as typing and reading. 

Interface pressure data were categorised into the following groups, dependent on the degree of unloading around the ischial tuberosity region between normal sitting and each movement using the pressure mapping Graphical User Interface (GUI) [24]: little or no change (0–25%), fair (26–50%), moderate (51–75%), and large or complete (76–100%). 

*Strand B.* A Shapiro-Wilk's test revealed that raw data for the variables angle of trunk tilt, DI, and CA during periods of normal sitting and periods of forward reach were normally distributed; however, data for the PPI (right and left ischial tuberosity) were not. Therefore, repeated measures ANOVA was used to further investigate the normally distributed data, and skewed data were interpreted using the Friedman Test and post hoc analysis using individual Wilcoxon Signed Rank Tests.

The relationship between trunk angle and interface pressure during repositioning movements in both strands was determined by a simple regression model.

## 3. Results

All 14 participants completed Strand A, 11 completed Strand B, and 13 completed the self-report questionnaire. 

*Strand A.* The frequency and type of movements performed by participants during the one hour period varied considerably (Table 2). One participant executed 28 movements, and three participants performed no movements (range 0–28, median 5 movements) during the 1 hour period. The majority of movements performed (30%) were categorised as task related movements. These included slight body movements to read the computer screen, talk to the researcher, type, and perform other automatic body functions/movements such as coughing and leg spasms.

Omitting the movements categorised as other or task related movements, participants performed a total of 49 movements (range per participant 0–12 movements, median 3 movements). No participants adhered to national recommendations of performing pressure relieving movements every 15 minutes [11]. However, 42.86% of participants did perform at least 4 pressure relieving movements during the 1 hour period. On average, the first nontask related movement was performed after 26 minutes and 37 seconds, and the majority of movements (71.4%) were held for less than 20 seconds, which is highly unlikely to be beneficial in terms of tissue reperfusion.

**Figure 2:**
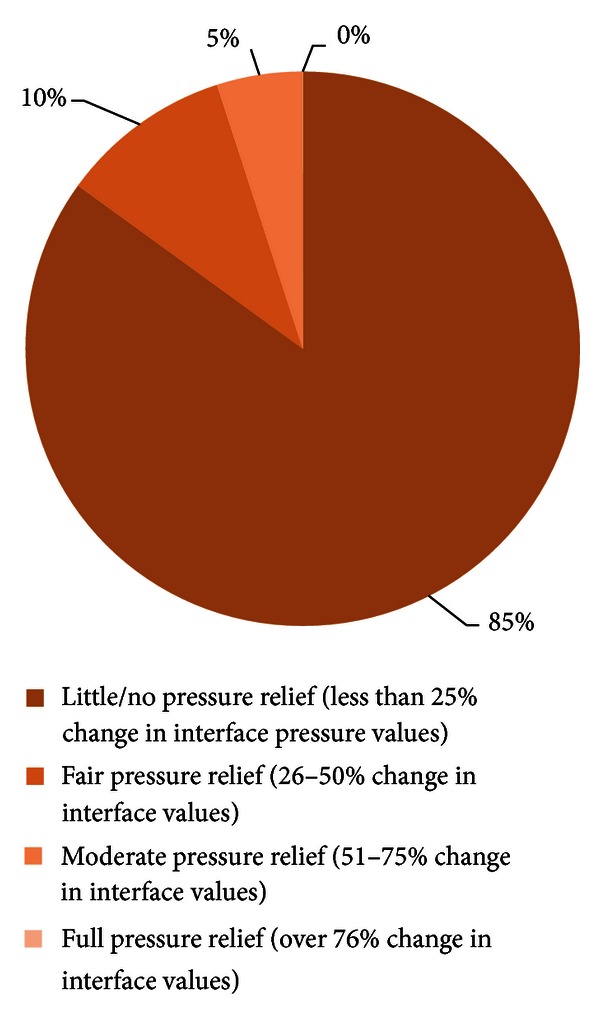
Effectiveness of repositioning movements performed during Strand A on seating interface pressures.

No movement performed over the 1 hour period was categorised as relieving 75–100% of interface pressures around the vulnerable ischial area, despite five of the participants performing “push-up” pressure reliefs during this time. The majority of all movements performed (84.4%) were categorised as yielding less than 25% reduction in interface pressures when compared to normal sitting. Thus, the effectiveness of the majority of weight shifts performed in terms of pressure relief was low (Figure 2).

A simple regression model, used to assess the ability of angle of trunk tilt to predict change in interface pressure using the parameters DI, PPI, and CA, showed that a 1% increase in trunk angle was associated with a minimal change in the parameters, indicating a weak relationship. 

*Strand B.* A one way repeated measures ANOVA revealed that reaching forward by 150% of arm length during a computer activity significantly reduced DI (*P* < 0.05) and angle of trunk tilt (*P* < 0.05) compared to normal sitting. However, compared to normal sitting, reaching forward by 150% did not significantly affect CA.

A Friedman Test showed that reaching forward by 150% of arm length significantly reduced PPI for both the right and left ischial tuberosity regions compared to normal sitting (*P* < 0.001) (mean PPI for the right ischial tuberosity decreased from 86.14 mmHg to 41.61 mmHg, and mean PPI for the left ischial tuberosity decreased from 81.55 mmHg to 38.89 mmHg). This represents a reduction in interface pressure of approximately 52% as a result of the 150% forward lean. Post hoc testing using individual Wilcoxon signed rank tests confirmed these results (*P* < 0.05). 

However, despite the angle of trunk tilt changing by 26.6° on average between normal sitting and reaching forward by 150%, a simple regression model showed a weak correlation between DI, CA, PPI, and angle of trunk tilt. A 1% increase in trunk angle was associated with a negligible change in DI, CA, and PPI, indicating a weak relationship. 

### 3.1. Participant Self-Report Questionnaire

Ten participants (77%) reported that they had never experienced a pressure ulcer, whilst three participants (23%) had a history of pressure ulceration. Of those participants who had experienced a pressure ulcer, the anatomical locations included: calves and heel, buttocks, and hip.

The majority of participants (*N* = 12) reported that they used a combination of traditional repositioning methods. The frequency of traditional repositioning movements reported varied considerably among participants, ranging from zero to approximately 60 daily, with the most popular method being a forward lean.

Seven participants (54%) found reaching forward by 150% during the computer activity to have been an achievable task to perform, rating the task as very easy (*n* = 1), easy (*n* = 4), or slightly difficult (*n* = 2). In contrast, three of the participants (23%) found this to be “difficult,” with one participant reporting that the reach was “too far” and “not comfortable.” Three of the fourteen participants were unable to complete Strand B, as their trunk stability would not sustain the forward reaching activity.

The majority of participants (*n* = 11; 85%) concluded that it would be beneficial to incorporate pressure reliefs within an activity, such as computer use. Likewise, the majority of participants (*n* = 10; 77%) also considered that incorporating pressure reliefs within an activity would make them easier to perform when compared to traditional pressure reliefs.

## 4. Discussion

The first aim of this study was to investigate the current pressure relieving behaviours of SCI individuals during everyday computer use. During a 1-hour computer activity, the number of seated movements varied considerably across participants. However, during this time the most frequently adopted position was a normal sitting position. These results are agreeable with able bodied computer users, reported to favour the reclined sedentary “normal sitting” posture when working intensively at the computer [25, 26].

Despite spending the majority of the working day in a “normal sitting posture,” able bodied office workers have been found to change seated position approximately once per minute during a thirty-minute computer activity [25]. Though mainly task related movements, this is in stark contrast to the current study which found that SCI subjects moved on average every 286.6 seconds (approximately every 5 minutes), and three participants performed no movements at all within the one hour period.

Close examination of the effectiveness of movements performed within Strand A of the current study, in terms of percentage unloading of the ischial tuberosities, showed that the majority of movements performed were ineffective in terms of pressure relief. Only occasionally (4.9% of all movements performed) did the movements generate “moderate” unloading of 51–75% reduction in interface pressures. Hence, although participants would have considered that seated movements were reducing interface pressures, analysis from pressure mapping revealed that these movements were ineffective for pressure relief.

Conversely, it could be argued that as interface pressure measurements were averaged over the duration of a movement from the initial posture change until a different posture was achieved, and brief episodes of complete unloading lasting 1-2 seconds may have been masked. However, it has been reported that tissue reperfusion rates for SCI population can take up to 300 seconds (5 minutes) [27]; therefore, it is unlikely that any minimal durations of complete unloading during a movement would have had any beneficial effect on tissue health in terms of reperfusion.

As the majority of movements performed (84.8%) produced minimal change in interface pressures, it can be concluded that the participants did not achieve the recommended four pressure relieving movements per hour [11], thus putting themselves at increased risk of tissue damage. These findings are comparable with previous research which found that, on average, most wheelchair users only reposition every 1-2 hours [12, 13, 28].

Despite the varying frequency of movements performed during Strand A, the effectiveness of the movements performed remained of low value in terms of pressure relief. Furthermore, results from the questionnaire revealed that participants believed they performed many pressure relieving movements throughout their average day. Considering the low pressure relieving value of the movements performed under observation, it would seem reasonable to question the worth of these movements currently being performed in “real life” as a preventative strategy for ulceration. 

The second aim of this study was to investigate the application of an ergonomically adapted computer-based activity to reduce interface pressure. Strand B of this study found that incorporating a 150% forward reach into a computer activity significantly decreased interface pressure, by approximately 52%, at the ischial region. Hence, reaching forward by 150% of arm length would be beneficial for reducing interface pressures for those at risk of tissue damage. Results are compatible with those of previous studies, which showed that pressure relief occurred as a result of leaning forward [29, 30] and during reaching activities [31–33].

Previous research reported that muscle and soft tissue deformations at the ischial tuberosity region decreased during a 40° forward lean (74%, 64%, resp.) compared to a 20° forward lean, which slightly increased tissue deformations (79%, 67%, resp.) for able bodied subjects [29]. Likewise, leaning forward by 45° produced the largest decreases in maximum pressure for children with myelomeningocele and able bodied comparisons [30]. Conversely, this study found that for a population of SCI participants, a forward reach of 150% arm length, which produced an average change in trunk angle of 24° (range 15°–35°) was enough to significantly reduced interface pressures. 

It should be noted, however, that three of the fourteen participants could not complete Strand B of the study due to poor trunk stability. A further three participants found the task to be either “difficult” or “very difficult.” Furthermore, researchers observed that the forward reaching movement patterns performed by the SCI participants differed considerably from those of able bodied or other disabled people. Shoulder protraction was greatly increased in an effort to limit the amount of pelvic movement required, to help overcome trunk stability issues. Despite this, responses from the majority who completed the questionnaire welcomed the idea of integrating weight shifts within a daily activity and believed that this would make pressure relieving movements easier to perform. 

However, although evidencing positive results for a 150% reach reducing interface pressures, this reduction was found to weakly correlate with a change in trunk angle. Therefore angle of trunk tilt is not a reliable indicator of interface pressure unloading and should not be used as an assessment component within clinical practice.

Repositioning, whether performed independently by clients or with assistance from healthcare professionals or carers, is an important part of an effective pressure ulcer prevention strategy, with the aim of redistributing high interface pressure from the bony ischial tuberosity region to other less vulnerable areas [11]. Healthcare professionals advocate leaning forward as a form of pressure relief; however, no specifications for the magnitude of forward lean required to redistribute pressure at the seating interface are currently recommended in guidelines. 

Repositioning is a powerful and practical measure that is within the domain and control of all healthcare professionals [34], including nurses and occupational therapists who should encourage clients at risk of pressure ulceration to perform pressure relieving movements frequently. In addition, the incorporation of pressure relieving movements into daily activities should be explored in an effort to improve client concordance with national pressure relieving recommendations [11].

## 5. Study Limitations

A limitation of this study was the small sample size (*N* = 14), which may affect generalisability of the results and may have affected the relationship predicted by a simple regression model between the angle of trunk tilt and interface pressures. It could also be argued that “normal sitting behaviour” was recorded over a relatively short duration, thus the results for movement frequency during the 1 hour period may be over inflated. Recording over a longer period of time may present different results of sitting behaviours within this population. Additionally, the visual demands of the computer task during strand A may have played a role in the frequency of movements performed [35].

Furthermore, as interface pressure measurements were averaged over the duration of a movement from the initial posture change until a different posture was adopted, brief episodes of complete unloading lasting 1-2 seconds may have been masked. However, it is likely that such a minimal duration of complete unloading would have any beneficial effect on tissue health in terms of reperfusion.

During strand B, the varying methods of reaching, including shoulder protraction and rotation, may have confounded results. It could also be argued that the presence of the researchers throughout the study may have skewed results; however, the exact nature of the study was not disclosed to participants in an effort to minimise a Hawthorne effect from occurring. 

## 6. Conclusion

This study investigated the current pressure relieving behaviours of SCI individuals during everyday computer use, and explored the application of an ergonomically adapted computer-based activity to reduce interface pressure. 

During a 1-hour computer activity, no participants adhered to national recommendations of performing pressure relieving movements as frequently or persistently as every 15 minutes [11]. It is of note that the majority of movements performed were held for less than 20 seconds, which is highly unlikely to be beneficial in terms of tissue reperfusion.

Indeed, the effectiveness of these seated movements in practice for reducing interface pressures was found to be low, therefore questioning the pressure relieving value of currently recommended movements as a preventative strategy for ulceration. Thus, further work is needed to investigate other methods of improving performance and concordance with repositioning methods among at risk populations. 

This study has shown that activity that incorporates a forward reach of 150% was capable of significantly reducing interface pressures in the ischial region.

Redistributing high interface pressure away from the bony ischial tuberosity region is important for reducing the risk of developing pressure ulcers during sitting. Healthcare professionals, including nurses and occupational therapists, should encourage clients at risk of pressure ulceration to perform pressure relieving movements frequently. Incorporation of pressure relieving movements, such as leaning/reaching forward, into everyday daily activities should be explored in an effort to improve client concordance with national pressure relieving recommendations [11].

## Figures and Tables

**Table 1:** Participant demographics.

Participant	Age	Sex	Inpatient/outpatient	Condition	Time since injury (months)	Height (cms)	Weight (kg)
001	23	Female	Inpatient	Paraplegic	3.5	174	Unknown
002	50	Male	Inpatient	Tetraplegic	4	180	84
003	26	Male	Inpatient	Poly neuropathy (tetraplegic)	1	183	66.5
004*	60	Male	Inpatient	Paraplegic	3	171.5	89.5
005*	40	Male	Inpatient	Tetraplegic	39	191	106.1
006	40	Female	Inpatient	Tetraplegic	3.5	170	79.4
007	62	Male	Outpatient	Paraplegic	9	173	92.1
008*	53	Male	Outpatient	Paraplegic	105	178	82.6
009	36	Male	Outpatient	Paraplegic	54	185	104.8
010	55	Male	Outpatient	Paraplegic	12	175	123.8
011	49	Male	Inpatient	Paraplegic	3	183	82.6
012	34	Male	Outpatient	Tetraplegic	324	155	Unknown
013	44	Male	Outpatient	Tetraplegic	29	183	85.7
014	48	Male	Inpatient	Paraplegic	1	173	69.9

*Participants who completed Strand A only.

**Table 2:** Type and quantity of movements performed during Strand A.

Participant	Normal sitting	Forward lean	Push-up	Left lean	Right lean	Other/task related movement	Total movements (including normal sitting)
001	15	12	0	0	0	7	34
002	11	4	2	1	6	15	39
003	4	1	0	0	0	2	7
004	6	0	1	0	0	5	12
005	1	0	0	0	0	0	1
006	3	2	0	0	1	1	7
007	6	0	5	0	0	0	11
008	3	0	0	1	0	4	8
009	8	2	0	1	2	9	22
010	5	0	1	1	0	4	11
011	1	0	0	0	0	0	1
012	6	1	0	2	1	5	15
013	1	0	0	0	0	0	1
014	2	0	1	1	0	2	6

Total movements	72	22	10	7	10	54	175

Percentage of movements	41%	13%	6%	4%	6%	30%	100%
